# The E-cadherin repressor Snail is associated with lower overall survival of ovarian cancer patients

**DOI:** 10.1038/sj.bjc.6604115

**Published:** 2007-11-20

**Authors:** K Blechschmidt, S Sassen, B Schmalfeldt, T Schuster, H Höfler, K-F Becker

**Affiliations:** 1 Institute of Pathology, Technical University of Munich, Trogerstrasse 18, Munich D-81675, Germany; 2 Department of Obstetrics and Gynecology, Technical University of Munich, Klinikum rechts der Isar, Ismaningerstrasse 22, Munich D-81675, Germany; 3 Institute of Medical Statistics and Epidemiology, Technical University of Munich, Ismaningerstrasse 22, Munich D-81675, Germany; 4 Institute of Pathology, GSF-National Research Centre for Environment and Health, Neuherberg D-85764, Germany

**Keywords:** Snail, E-cadherin, epithelial–mesenchymal transition, ovarian cancer, overall survival

## Abstract

Epithelial ovarian cancer is the leading cause of death among female genital malignancies. Reduced expression of the cell adhesion molecule E-cadherin was previously shown to be associated with adverse prognostic features. The role of the E-cadherin repressor Snail in ovarian cancer progression remains to be elucidated. We analysed formalin-fixed and paraffin-embedded specimens of 48 primary ovarian tumours and corresponding metastases for expression of E-cadherin and Snail by immunohistochemistry. We found a significant correlation between E-cadherin expression in primary cancers and their corresponding metastases (*P*<0.001). This correlation was found for Snail expression as well (*P*<0.001). There was a significant (*P*=0.008) association of reduced E-cadherin expression in primary ovarian cancer with shorter overall survival. Similarly, Snail expression in corresponding metastases (*P*=0.047) was associated with reduced overall survival of the patients. Additionally, the group of patients showing reduced E-cadherin and increased Snail immunoreactivity in primary tumours and corresponding metastases, respectively, had a significantly higher risk of death (*P*=0.002 and 0.022, respectively) when compared to the patient group with the reference expression profile E-cadherin positive and Snail negative. Taken together, the results of our study show that the E-cadherin repressor Snail is associated with lower overall survival of ovarian cancer patients.

Epithelial ovarian cancer is the second most common gynaecological cancer and the leading cause of death among female genital malignancies in the developed world (Parkin *et al*, 2005; Sankaranarayanan and Ferlay, 2005). More than two-thirds of patients with ovarian cancer are diagnosed with advanced-stage disease, because ovarian cancer is often asymptomatic in its early stages (Ozols, 2005). The degree of peritoneal dissemination is related to the poor prognosis in patients with advanced-stage ovarian cancer. The molecular mechanisms that allow ovarian cancer cells to detach from the primary tumour, invade the peritoneal surfaces, and regrow at this site are not yet well understood. Nevertheless, it is known that epithelial–mesenchymal transition (EMT) is an important mechanism during early steps of tumour progression where tumour cells disseminate from the primary tumour (Thiery and Chopin, 1999; Thiery, 2002, 2003; Gotzmann *et al*, 2004). Predominantly, EMT is a key process in normal embryonic development by which epithelial cells acquire a fibroblastoid morphology, accompanied by the loss of epithelial and gain of mesenchymal markers (Hay, 1995; Thiery and Chopin, 1999; Thiery, 2003). The process of acquisition of the invasive phenotype by epithelial tumours can be regarded as a pathologic version of the EMT of embryogenesis.

Expression of the homophilic Ca^2+^-dependent cell adhesion molecule E-cadherin is lost during EMT. As a key regulator of the differentiated epithelial phenotype, E-cadherin plays a critical role in the suppression of tumour invasion, and its function is required for the maintenance of stable adherens junctions and epithelial cell polarity (Takeichi, 1991; Perez-Moreno *et al*, 2003). Occurrence of altered E-cadherin expression has been correlated with dedifferentiation, increased risk of local invasion and metastatic disease, and recurrence and poor prognosis in a variety of carcinomas, for example, breast, uterine cervix, or gastric carcinomas (Moll *et al*, 1993; Fujimoto *et al*, 1997; Jawhari *et al*, 1997; Jeffers *et al*, 1997; Huiping *et al*, 2001). Previous studies have shown that reduced expression of E-cadherin in ovarian cancer is associated with the invasive phenotype, advancing tumour stage, lower 5-year survival rate, and poor recurrence-free survival (Faleiro-Rodrigues *et al*, 2004; Imai *et al*, 2004; Marques *et al*, 2004; Voutilainen *et al*, 2006) but not much is known about the underlying mechanisms of E-cadherin downregulation.

A regulator of E-cadherin expression is the transcription factor Snail, a member of the Snail superfamily of zinc-finger transcription factors, which was first identified in *Drosophila melanogaster* (Grau *et al*, 1984; Nieto, 2002). Snail mediates events in mesoderm, neuroectoderm, and other organ development in the embryo but is also an important effector of the process of invasiveness and tumorigenecity (Hemavathy *et al*, 2000). Snail is considered as a key regulator of EMT as it represses the transcription of E-cadherin by binding to E-box elements found in the proximal E-cadherin promoter, thereby triggering a complete EMT with the acquisition of invasive and tumorigenic properties (Batlle *et al*, 2000; Cano *et al*, 2000; Peinado *et al*, 2004; De Craene *et al*, 2005). In humans, Snail mRNA expression has been detected in biopsies or resected tissue samples from patients with breast cancer (Blanco *et al*, 2002), gastric cancer (Rosivatz *et al*, 2002), hepatocellular carcinomas (Jiao *et al*, 2002), oral squamous cell carcinoma (Yokoyama *et al*, 2001), and ovarian carcinoma (Elloul *et al*, 2006). Because Snail is significantly regulated at the protein level, we decided to analyse Snail immunoreactivity in ovarian cancer. Our recently established Snail-specific antibody allows a direct cellular comparison between E-cadherin downregulation and endogenous nuclear Snail expression at the protein level in cancer tissues (Rosivatz *et al*, 2006; Blechschmidt *et al*, 2007).

The aim of our study was to elucidate the role of the transcription factor Snail in E-cadherin downregulation and cancer progression in ovarian carcinomas.

## MATERIALS AND METHODS

### Tissue samples

A total of 51 patients who had undergone primary surgery for newly diagnosed advanced-stage (FIGO IIIC and IV) ovarian cancer between 1998 and 2001 at the University Hospital of the Technical University of Munich were eligible for this retrospective analysis. Exclusion criteria were a second malignancy or chemotherapy or radiotherapy within the last 6 months prior to surgery. Follow-up data were available for all patients, with a median follow-up time of 55 months.

Formalin-fixed and paraffin-embedded specimens of the primary ovarian tumours and corresponding metastases were immunohistochemically analysed for expression of E-cadherin and Snail. In 47 cases, specimens of the primary tumour as well as the corresponding metastases were analysed, in one case only the primary tumour was available, and in three cases only specimens of metastases were analysed. Metastases were located in the peritoneum (*n*=17), omentum (*n*=25), distant lymph nodes (*n*=6), and uterus (*n*=2).

### Immunohistochemistry

Sections (4 *μ*m) of formalin-fixed and paraffin-embedded material were analysed. For detection of E-cadherin- and Snail-specific immunoreactivity, sections were treated as follows: after standard pressure-cooker-based antigen retrieval with citric acid (pH 6.0) pretreatment, sections were incubated with either H_2_O_2_ (for E-cadherin) or normal goat serum (for Snail).

The sections were incubated with either a monoclonal anti-E-cadherin antibody (1 : 1500) (clone 36; Transduction Laboratories, Lexington, KY, USA) at room temperature for 1 h or a monoclonal anti-Snail antibody (1 : 20) (hybridoma supernatant Sn9H2; Rosivatz *et al*, 2006) at room temperature for 2 h. Both antibodies were detected using the avidin–biotin complex peroxidase method (ABC Elite Kit; Vector, Burlingame, CA, USA). Final staining was developed with the Sigma FAST DAB peroxidase substrate kit (Sigma, Deisenhofen, Germany). Haemalaun was used for counterstaining. For the analysis of E-cadherin immunoreactivity, adjacent endometrium, intestinal mucosa, or non-tumorous ovarian surface epithelium was used as an endogenous positive control. Sections of archival placental tissue were used as a positive control for Snail immunoreactivity (Rosivatz *et al*, 2006).

### Immunohistochemical evaluation

Expression of E-cadherin and Snail was assessed using a semiquantitative scoring system, ranging from 0, 1+, 2+, and 3+.

E-cadherin expression was scored as follows:

- 0, no immunoreactivity or immunoreactivity of <10% of tumour cells
- 1+, low-intensity immunoreactivity of ⩾10% of tumour cells
- 2+, medium-intensity immunoreactivity of ⩾10% of tumour cells
- 3+, high-intensity immunoreactivity of ⩾10% of tumour cells

All cases were summarised into two groups, showing preserved E-cadherin expression (score 3+) or reduced E-cadherin expression (scores 0, 1+, and 2+).

Snail expression was evaluated as following:

- 0, no immunoreactivity or immunoreactivity of <1% of tumour cells
- 1+, immunoreactivity of 1% of tumour cells
- 2+, immunoreactivity of 2–5% of tumour cells
- 3+, immunoreactivity of >5% of tumour cells

Snail staining was graded as positive only when nuclear staining was detectable.

All cases were summarised into two groups, negative for Snail expression (score 0) or positive for Snail expression (scores 1+, 2+, and 3+).

### Statistical analysis

Continuous variables are reported as median with range, and categorical data are expressed as frequencies and percentages. *χ*^2^-test and if appropriate Fisher Exact test were applied to test for bivariate associations of categorical parameters. Logistic regression was used to perform multivariate investigations of binary-response variables. Analysis of survival times was performed using the method of Kaplan–Meier, and comparison of survival between patient subgroups was done by Log-rank test and Cox proportional hazard regression. Hazard ratios and median survival were reported with 95% confidence intervals. All tests were performed two-tailed at a 5% level of significance. Bonferroni adjustment of *α*-error rate was considered in case of multiple comparisons, in particular performing subgroup analysis. Analyses were conducted using SPSS version 14.0 (SPSS Inc., Chicago, IL, USA).

## RESULTS

**Figure 1:**
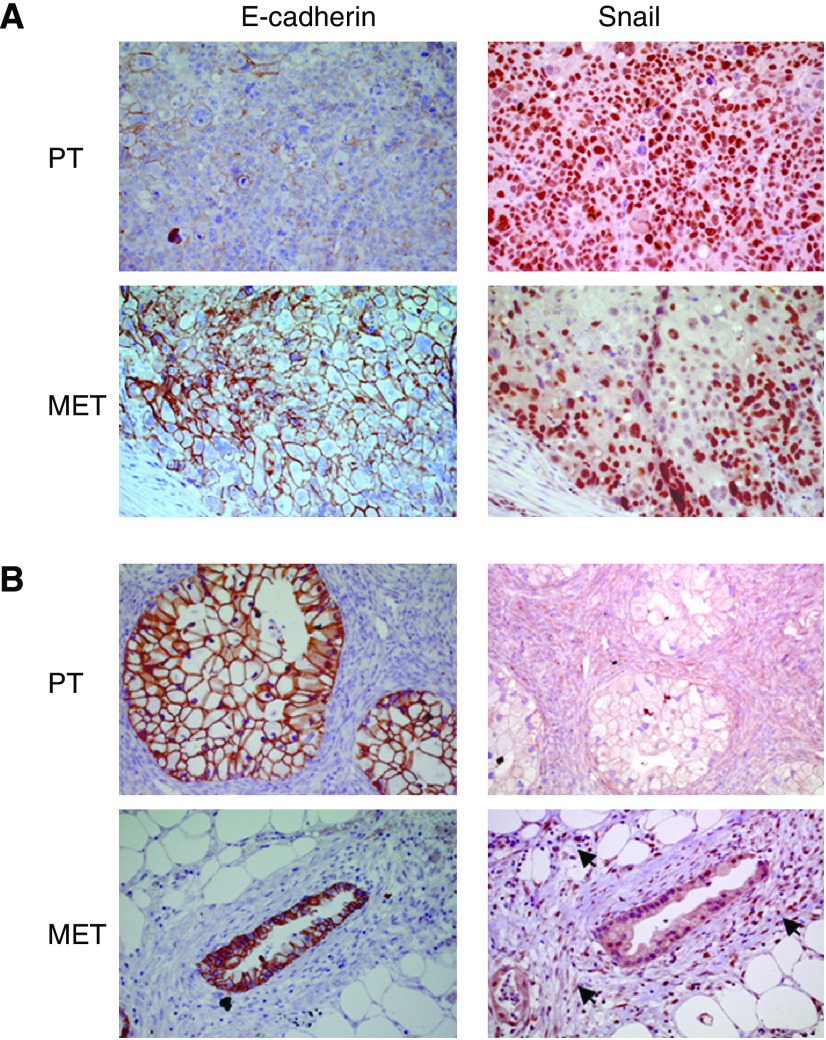
(**A**, **B**) Consecutive immunostainings for E-cadherin and Snail of two ovarian cancer cases showing the primary tumours and the corresponding metastases. (**A**) An ovarian cancer case showing reduced E-cadherin immunostaining in the primary tumour (1+), while E-cadherin immunoreactivity is preserved in the corresponding metastasis (3+). Snail immunoreactivity is positive (3+) for both, primary tumour and metastasis (immunoperoxidase staining, × 200). (**B**) An ovarian cancer case showing preserved E-cadherin immunostaining (3+) in primary tumour and corresponding metastasis. Snail immunoreactivity is negative (0) in the primary tumour, whereas it is positive (1+) in the corresponding metastasis. Note that tumour-associated stromal cells also show nuclear staining for Snail (indicated by arrowheads) (immunoperoxidase staining, × 200). PT, primary tumour; MET, corresponding metastasis.

A total of 48 specimens of primary tumours and 50 specimens of metastases of ovarian carcinomas were examined for E-cadherin and Snail immunoreactivity, of which 47 cases were matched pairs of primary tumours and their corresponding metastases (Figure 1). A summary of the clinicopathological characteristics of the patients is given in Table 1.

### E-cadherin expression in primary and metastatic ovarian cancer

E-cadherin expression was preserved (score 3+) in 75% of primary ovarian cancers and 78% of the corresponding metastases. A reduced E-cadherin immunoreactivity (score 0, 1+, and 2+) was found in 25% of primary tumours and 22% of metastases (Table 2A).

There was a significant correlation between E-cadherin expression in ovarian cancers and their corresponding metastases (*P*<0.001). Out of 35 primary tumours with preserved E-cadherin expression, 33 (94.3%) showed preserved expression in the corresponding metastases. Out of 12 primary tumours with reduced E-cadherin expression, 8 (66.7%) showed reduced expression in the corresponding metastases (Table 3). There was no difference in E-cadherin expression in various metastatic locations.

No association was found between E-cadherin immunoreactivity and clinicopathological factors including patient age, tumour grade, histological subtype, and FIGO stage in primary ovarian cancers, nor in the corresponding metastases. These results were confirmed by multivariate analysis considering E-cadherin immunoreactivity in primary cancers and metastases, respectively, and TNM status, tumour grade, histological subtype, and FIGO stage. No statistical significance was found for multivariate analysis as well.

### Snail expression in primary and metastatic ovarian cancer

Expression of Snail (score 1+, 2+, and 3+) was detected in 37.5% of primary ovarian cancers and in 52% of corresponding metastases (Table 2B). There was a significant correlation between Snail expression in ovarian cancers and their corresponding metastases (*P*<0.001). Out of 17 primary tumours with positive Snail expression, 16 (94.1%) showed Snail expression in the corresponding metastases. Out of 30 primary tumours with negative Snail immunoreactivity, 21 (70.0%) were negative for Snail in the corresponding metastases (Table 3). There was no difference in Snail expression in various metastatic locations. No association was found between Snail expression and clinicopathological factors including patient age, tumour grade, histological subtype, and FIGO stage in primary ovarian cancers nor in the corresponding metastases. These results were confirmed by multivariate analysis considering Snail immunoreactivity in primary cancers and metastases, respectively, and TNM status, tumour grade, histological subtype, and FIGO stage. No statistical significance was found for multivariate analysis as well.

There was no statistically significant correlation between E-cadherin and Snail expression, neither in primary ovarian cancers nor in their metastases. Out of 36 primary tumours with preserved E-cadherin expression, 13 (36.1%) showed positive Snail and 23 (63.9%) negative Snail immunoreactivity (*P*=0.743). Out of 39 metastases with preserved E-cadherin expression, 21 (53.8%) showed positive Snail and 18 (46.2%) negative Snail immunoreactivity (*P*=0.623).

### E-cadherin and Snail expression and overall survival

**Figure 2:**
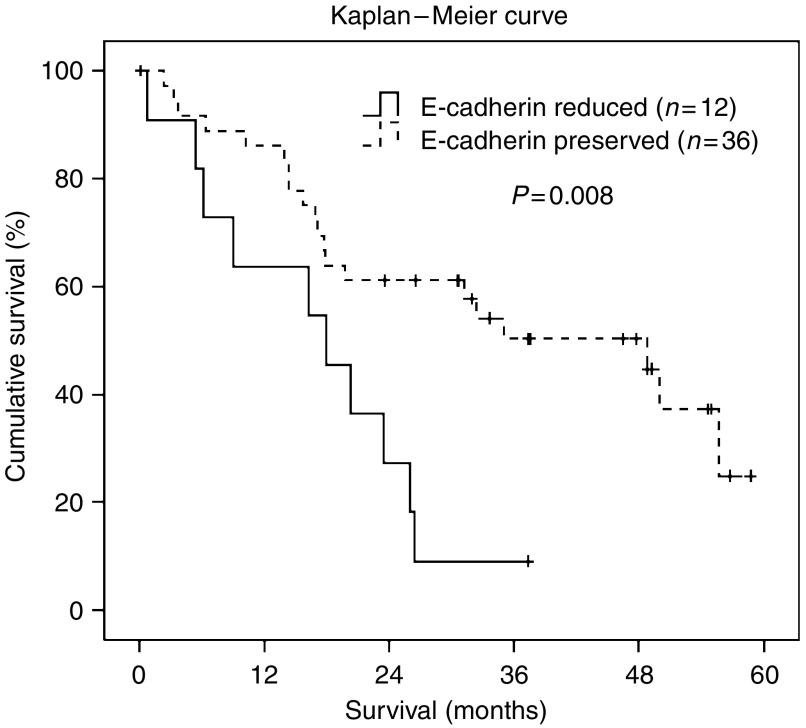
Kaplan–Meier survival curve for ovarian carcinoma patients according to E-cadherin immunoreactivity in primary ovarian cancers. Discontinuous line represents preserved E-cadherin immunoreactivity (3+). Continuous line represents reduced E-cadherin immunoreactivity (2+, 1+, and 0). Patients with reduced E-cadherin immunoreactivity had a significantly shorter overall survival (*P*=0.008).

There were significant differences in patients’ overall survival with regard to E-cadherin expression in primary ovarian cancer (*P*_(log-rank)_=0.008; significant according to the adjusted significance level) (Figure 2). Patients with a reduced E-cadherin expression (*n*=12) had a median overall survival of 17.9 months (95% CI: 5.8–30.0) compared to 48.8 months (95% CI: 24.0–73.6) in patients with preserved E-cadherin expression (*n*=36). The corresponding hazard ratio was 2.82 (95% CI: 1.3–6.3). In metastases, no significant differences were found in overall survival when examining E-cadherin expression.

**Figure 3:**
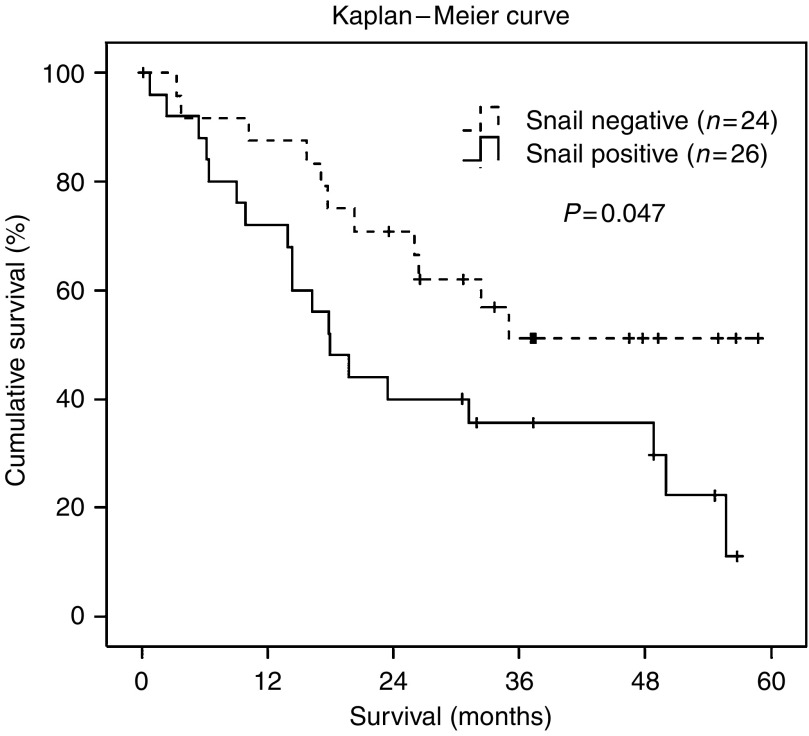
Kaplan–Meier survival curve for ovarian carcinoma patients according to Snail immunoreactivity in metastases of ovarian cancer. Discontinuous line represents negative Snail immunoreactivity (0). Continuous line represents positive Snail immunoreactivity (1+, 2+, and 3+). Patients with positive Snail immunoreactivity had a significantly shorter overall survival (*P*=0.047).

There was a borderline significant difference in the overall survival of patients with positive Snail expression in metastases of ovarian cancer compared to patients with negative Snail expression (*P*_(log-rank)_=0.047) (Figure 3), whereas these differences were not found in primary tumours. Patients with positive Snail expression in their metastases (*n*=26) had a lower median overall survival of 17.9 months (95% CI: 12.2–23.7) with a hazard ratio of 2.10 (95% CI: 1.0–4.4). The median overall survival of patients with Snail negative metastases (*n*=24) has not yet been reached at 55 months of median follow-up time.

To analyse the patients’ overall survival with regard to different combinations of E-cadherin and Snail expression profiles, we divided the patients into four different groups, which are shown in Tables 4A and 4B. The comparison of survival between the different groups was done by multivariate Cox proportional hazard regression adjusted for patient age. Since normal non-cancerous epithelial tissue is characterised by preserved E-cadherin and negative Snail expression, this expression profile served as a reference standard. When analysing the protein expression profile in primary ovarian tumours with regard to patient survival, we found that only patients with the expression profile ‘E-cadherin reduced and Snail positive’ showed a significantly (*P*=0.002; significant according to the adjusted significance level) higher risk for the occurrence of death with a hazard ratio of 5.91 (95% CI: 1.9–18.0) when compared to the reference group (Table 4A). In case of the corresponding metastases, a significantly (*P*=0.025) higher risk for the occurrence of death was seen in patients with the same expression profile of ‘E-cadherin reduced and Snail positive’ with a hazard ratio of 4.26 (95% CI: 1.2–15.2) when compared to the reference group (Table 4B). Taken together, a total of eight patients showed this profile of reduced E-cadherin and positive Snail expression in either the primary tumour (*n*=5) or metastases (*n*=5). (Two of these patients showed this profile in both their primary tumor and metastases.) Out of these eight patients, five (62.5%) were serous carcinomas, two (25%) endometrioid, and one (12.5%) mucinous carcinoma. This distribution of histological subtypes reflects their statistical frequency in the study population (see Table 1).

## DISCUSSION

In the current study, we analysed the active, that is, nuclear-localised, Snail protein and its target E-cadherin by immunohistochemistry in a series of primary ovarian carcinomas and their corresponding metastases. The results show a significant association between reduced E-cadherin expression in primary ovarian cancer and shorter overall survival of the patients. Similarly, positive Snail expression in the corresponding metastases was correlated with reduced overall survival. Interestingly, for both E-cadherin and Snail, we observed no significant difference in expression between primary tumours and their corresponding metastases.

The cell adhesion molecule E-cadherin has already been described to be involved in tumour dedifferentiation and poor recurrence-free survival in ovarian cancer (Imai *et al*, 2004; Voutilainen *et al*, 2006). We showed that reduced E-cadherin immunoreactivity in primary ovarian tumours was significantly (*P*=0.008) associated with shorter overall survival.

A key regulator of E-cadherin expression is the zinc-finger transcription factor Snail, a master molecule of EMT (Cano *et al*, 2000). Up to now, more than 2000 cases from at least nine different tumour types reported in more than 21 studies have been analysed for Snail expression, including carcinomas from breast, stomach, colon, liver, ovary, oesophagus, head and neck, and endometrium, and synovial sarcomas (Becker *et al*, 2007). Elloul *et al* (2006) recently analysed the expression of E-cadherin and its transcriptional regulators in ovarian cancer. They evaluated the expression of the repressors Snail, Slug, and SIP1 in fresh non-fixed material of ovarian primary carcinomas, solid metastases, and malignant peritoneal and pleural effusions by reverse transcription (RT)-PCR and western blot. The authors reported positive Snail expression in the majority of the analysed tumour specimens. Snail mRNA expression was detected in 93% (38/41) primary tumours, 93% (14/15) metastases, and 87% (68/78) effusions. Snail protein expression determined by Western blot analysis was found in 100% (30/30) primary tumours, 100% (10/10) metastases, and 97% (72/74) effusions. The authors found that mean expression levels of Snail protein were lower in effusions, with expression of 17% of control levels in effusions compared to 118% in primary tumours and 127% in metastases (Elloul *et al*, 2006). These discrepancies between Snail mRNA and protein expression levels may partly be explained by potential posttranslational regulation mechanisms. Besides being tightly regulated at the transcriptional level, Snail's activity is also influenced by its subcellular localisation. In ovarian tumour cells derived from effusions, Elloul *et al* (2006) found that Snail protein was exclusively localised in the cytoplasm, which may reflect an inactive form of the protein (Dominguez *et al*, 2003). Snail can cycle between the nucleus and the cytosol by virtue of a nuclear export sequence. At least two kinases, glycogen synthase kinase-3*β* and p21-activated kinase-1, are known to govern Snail's localisation (Dominguez *et al*, 2003; Zhou *et al*, 2004; Yang *et al*, 2005). In addition, the zinc-finger transporter LIV-1 seems to be involved in this level of regulation, as shown for zebrafish (Yamashita *et al*, 2004). The complexity of Snail's functional activation makes clear that it is a shortcoming of Western blot analysis that only total protein expression is reflected, which does not necessarily correlate to the amount of active Snail.

In the current study, we analysed the expression of nuclear-localised, active Snail. We found that 37.5% of the primary tumours and 52% of the corresponding metastases showed a positive nuclear staining for Snail. In addition to Snail-positive carcinoma cells, we also found tumour-associated stromal cells showing a positive immunoreactivity for Snail. Snail-positive stromal cells have been described previously by us (Blechschmidt *et al*, 2007) and by Franci *et al* (2006) in endometrial and colon cancer, respectively. This raises the question whether these Snail-positive stromal cells represent former tumour cells that have undergone mesenchymal transition. On the other hand, a *de novo* expression of Snail in tumoral stroma might suggest a key role for stromal cells in promoting tumour progression. The role of Snail in tumour-associated stromal cells needs to be established in additional studies.

We found a significant correlation between Snail expression in primary ovarian tumours and their corresponding metastases (*P*<0.001). Cases (94.1%) with a positive Snail immunoreactivity in primary tumours were also Snail positive in the corresponding metastases. On the other hand, 70% of cases with Snail-negative primary tumours were also Snail negative in the corresponding metastases, yet 30% showed a positive Snail immunoreactivity. In previous studies, it was shown that Snail not only induces tumour invasion but also blocks the cell cycle and confers resistance to cell death (Vega *et al*, 2004). Our results indicate that these or other, yet unknown, features of Snail might be of special importance for the establishment or maintenance of metastases at the new invasion site in ovarian cancer, possibly leading to preserved Snail expression. Additionally, we demonstrated that positive Snail immunoreactivity in metastases of ovarian cancer was significantly associated with a lower overall survival of the patients. No association was found between Snail expression in primary ovarian cancer and survival.

There was no correlation between Snail immunoreactivity and other clinicopathological parameters, including patient age, tumour subtype, or grade of differentiation. The last might be due to the small numbers of low-grade and low-stage cases as discussed above. In the current study, there was no correlation between Snail upregulation and E-cadherin downregulation, neither in primary tumours nor in corresponding metastases. We found coexpression of E-cadherin and Snail in 36.1% of primary tumours and in 53.8% of metastases. These results are in accordance with previous studies on endometrial and colon cancer (Franci *et al*, 2006; Blechschmidt *et al*, 2007), although it is not yet understood why Snail expression does not lead to E-cadherin downregulation in these cases.

We also asked whether specific combinations of E-cadherin and Snail protein expression had a prognostic value. We found that a profile of reduced E-cadherin expression and nuclear Snail expression was associated with a significantly increased risk of death. Patients showing an ‘E-cadherin reduced and Snail positive’ profile, in either the primary tumours or corresponding metastases, respectively, had a 6-fold and 4.2-fold increased risk of death (*P*=0.002 and 0.022, respectively) when compared to the patient group with a ‘normal epithelial’ expression profile of ‘E-cadherin positive and Snail negative’.

Additionally, patients with a profile of preserved E-cadherin and positive Snail expression in metastases were at an increased risk of death (*P*=0.077), although this association was not statistically significant. Taken together, these observations indicate that Snail might be an independent prognostic factor for clinical outcome in ovarian cancer. Snail may be important for the establishment and maintenance of metastases in ovarian cancer, while loss of E-cadherin expression might be crucial in primary tumours, for example, for tumour invasion, both leading to an adverse clinical outcome for ovarian cancer patients.

This is the first study in which the subcellular expression of the E-cadherin repressor Snail has been analysed in a series of ovarian carcinomas. Our findings, although derived from a limited number of patients, form an important basis for future prospective studies. In conclusion, the results of our study show that Snail is associated with lower overall survival of ovarian cancer patients and provide new evidence for a role of Snail as a prognostic factor for adverse clinical outcome in ovarian cancer.

## Figures and Tables

**Table 1:** Clinicopathological characteristics of the patients (*n*=51)

**Characteristic**	***n***	**%**
*Age, years*		
Median	63	
Range	29–82	
		
*Histological grade*		
1	3	6
2	11	22
3	37	72
		
*FIGO stage*		
III	39	76
IV	12	24
		
*TNM staging*		
T		
1	3	6
2	2	4
3	46	90
		
*N*		
0	6	12
1	22	43
x	23	45
		
*M*		
0	38	74
1	11	22
x	2	4
		
*Histological type*		
Serous	39	76
Endometrioid	5	10
Others^a^	7	14
		
*End state*		
Dead	31	61
Alive	20	39

a Includes three mucinous, two Muellerian, one Brenner, and one clear cell.

**Table 2A:** E-cadherin expression in primary ovarian cancer and corresponding metastases

		**Primary tumours (*n*=48)^a^**		**Metastases (*n*=50)^a^**	
**E-cadherin**	**Score**	**(*n*)**	**(%)**	**(*n*)**	**(%)**
**Reduced**		**12**	**25.0**	**11**	**22.0**
	0	1	2.1	1	2.0
	1+	2	4.2	1	2.0
	2+	9	18.7	9	18.0
**Preserved**	3+	**36**	**75.0**	**39**	**78.0**

a For *n*=47 cases, matched pairs of primary tumours and their corresponding metastases were available for analysis.

Reduced, reduced E-cadherin expression defined by scores of 0, 1+, and 2+.

Preserved, preserved E-cadherin expression defined by a score of 3+.

*n*, number of patients.

The main categories of ‘reduced’ and ‘preserved’ (as compared to the subcategories of reduced expression) are shown in bold.

**Table 2B:** Snail expression in primary ovarian cancer and corresponding metastases

		**Primary tumours (*n*=48)^a^**		**Metastases (*n*=50)^a^**	
**Snail**	**Score**	**(*n*)**	**(%)**	**(*n*)**	**(%)**
**Negative**	0	**30**	**62.5**	**24**	**48.0**
**Positive**		**18**	**37.5**	**26**	**52.0**
	1+	11	22.9	17	34.0
	2+	4	8.3	6	12.0
	3+	3	6.3	3	6.0

a For *n*=47 cases, matched pairs of primary tumours and their corresponding metastases were available for analysis.

Negative, no expression of Snail defined by a score of 0.

Positive, positive Snail expression defined by scores of 1+, 2+, and 3+.

*n*, number of patients.

The main categories of ‘negative’ and ‘positive’ (as compared to the subcategories of positive expression) are shown in bold.

**Table 3:** Correlation of E-cadherin and Snail expression in primary ovarian cancer compared to the corresponding metastases^a^

	**E-cadherin (*n*)**	**Snail (*n*)**
Primary positive	35	17
Primary negative	12	30
Primary positive, metastasis positive	33 (94.3% of *n*=35)	16 (94.1% of *n*=17)
Primary positive, metastasis negative	2 (5.7% of *n*=35)	1 (5.9% of *n*=17)
Primary negative, metastasis negative	8 (66.7% of *n*=12)	21 (70.0% of *n*=30)
Primary negative, metastasis positive	4 (33.3% of *n*=12)	9 (30.0% of *n*=30)

a *n*=47 cases for which both the primary tumours and their corresponding metastases were available.

Primary, primary ovarian cancer.

Metastasis, corresponding metastases of primary ovarian cancers.

Positive, preserved immunoreactivity for E-cadherin (3+) or Snail positive (1+, 2+, and 3+).

Negative, reduced immunoreactivity for E-cadherin (0, 1+, and 2+) or Snail negative (0).

**Table 4A:** Comparison of survival in patient groups with different expression profiles of E-cadherin and Snail in primary ovarian cancer using multivariate Cox proportional hazard regression adjusted for patient age

			**95% CI for HR**	
**Primary ovarian cancer (*n*=48)**	***P***	**HR**	**Lower**	**Upper**
E-cadherin+/Snail− (*n*=23)^a^		1.00		
E-cadherin+/Snail+ (*n*=13)	0.590	1.28	0.521	3.150
E-cadherin−/Snail− (*n*=7)	0.165	2.13	0.733	6.191
E-cadherin−/Snail+ (*n*=5)	**0.002^#^**	5.91	1.973	18.036
Age (years)	0.890	1.01	0.973	1.032

CI=confidence interval; HR=hazard ratio for the occurrence of death; +=preserved immunoreactivity for E-cadherin (3+) or Snail positive (1+, 2+, and 3+); −=reduced immunoreactivity for E-cadherin (0, 1+, and 2+) or Snail negative (0).

a Was set as the reference group.

^#^Significant according to the adjusted significance level.

*n*, number of patients.

Bold value signifies *P*-value <0.05.

**Table 4B:** Comparison of survival in patient groups with different expression profiles of E-cadherin and Snail in metastases of ovarian cancer using multivariate Cox proportional hazard regression adjusted for patient age

			**95% CI for HR**	
**Metastases of ovarian cancer (*n*=50)**	***P***	**HR**	**Lower**	**Upper**
E-cadherin+/Snail− (*n*=18)^a^		1.00		
E-cadherin+/Snail+ (*n*=21)	0.069	2.35	0.936	5.884
E-cadherin−/Snail− (*n*=6)	0.313	1.90	0.547	6.601
E-cadherin−/Snail+ (*n*=5)	**0.025** ^#^	4.26	1.198	15.159
Age (years)	0.692	1.01	0.977	1.036

CI=confidence interval; HR=hazard ratio for the occurrence of death; +=preserved immunoreactivity for E-cadherin (3+) or Snail positive (1+, 2+, and 3+); −=reduced immunoreactivity for E-cadherin (0, 1+, and 2+) or Snail negative (0).

a Was set as the reference group.

*n*, number of patients.

Significant according to the adjusted significant level. Bold value signifies *P*-value <0.05.
